# Editorial: The challenge of immunity evaluation and immunotherapy in gynecologic and urologic oncology

**DOI:** 10.3389/fimmu.2023.1261229

**Published:** 2023-08-01

**Authors:** Ruirong Tan, Miao Liu, Yiguan Zhang, Rui Li

**Affiliations:** ^1^ Center for Organoids and Translational Pharmacology, Translational Chinese Medicine Key Laboratory of Sichuan Province, Sichuan Institute for Translational Chinese Medicine, Sichuan Academy of Chinese Medicine Sciences, Chengdu, China; ^2^ Department of Pathology, Brigham and Women’s Hospital, Harvard Medical School, Boston, MA, United States; ^3^ Department of Radiation Oncology, Radiation Oncology Key Laboratory of Sichuan Province, Sichuan Clinical Research Center for Cancer, Sichuan Cancer Hospital & Institute, Sichuan Cancer Center, Affiliated Cancer Hospital of University of Electronic Science and Technology of China, Chengdu, China

**Keywords:** immunity, immunotherapy, gynecologic oncology, protac, immune evaluation

## Introduction

In recent years, tumor immunotherapy, which modulates the body’s own immune system to fight against tumors, has become one of the important approaches in the treatment of malignant tumors. Immune checkpoint inhibitors, represented by immune checkpoint blockade agents, have significantly improved the five-year survival rate of advanced cancer patients and are considered a promising treatment modality for curing cancer (1). As the field of tumor immunotherapy continues to advance rapidly and is being applied more widely in clinical practice, various types of tumors, including gynecological tumors, have benefited from it. However, cancer immunotherapy is only effective for a subset of patients and is associated with issues such as drug resistance and adverse reactions. Therefore, determining strategies to improve the efficacy of immune checkpoint blockade (ICB) remains a major clinical need. Accurately identifying the population most likely to benefit from treatment has become a major challenge in tumor immunotherapy research.

## Identification of immune evaluation indicators in gynecological tumors

Currently, several biomarkers related to immunotherapy in gynecological cancers are being studied and have the potential to be used for clinical screening of treatment-beneficial populations (2). However, these biomarkers also have many limitations. Novel single-cell transcriptomic technologies provide strong support for the identification of relevant biomarkers. In the study of rare ovarian teratoma mechanisms, single-cell analysis has provided a comprehensive overview of programmed cell death and molecular-level evidence supporting the distribution of immune cells in different types of ovarian teratoma and the prognosis (Cao et al.). This has further improved our understanding of the tumor microenvironment. Recurrent or metastatic urological and gynecological tumors typically exhibit resistance to traditional cancer treatment methods, which is highly influenced by interethnic and interpopulation differences. Immunotherapy brings hope to these patients. However, although the scoring of the tumor microenvironment is an important predictor of ICB efficacy, the lack of systematic research is one of the challenges faced in gynecological tumor immunotherapy. In this regard, similarly, data at the single-cell level can help establish a new scoring system for tumors. Using this method, three key genes, IL1B, CST7, and ITGA5, were identified for the first time in different cervical cancer cell types and their correlation with the proliferative and invasive capabilities of cervical cancer cells (Yao et al.). This established an immune microenvironment scoring system for this type of tumor, providing guidance for future immunotherapy. However, with the widespread use of ICB, the issue of acquired resistance inevitably arises.

## Novel immunotherapy approaches

PD-1/PD-L1 is a promising target for inducing anti-tumor immune responses. Compared to other immune checkpoints, PD-1 has a broader expression range. PD-1 limits the activity of T cells in peripheral tissues during inflammatory responses by binding to its ligands, PD-L1 and PD-L2, thereby minimizing autoimmune reactions. PD-L1 expression levels are high in ovarian cancer and cervical cancer samples. In recent years, various anti-PD-1 and anti-PD-L1 drugs, such as pembrolizumab and atezolizumab, have been developed. Key trials such as KEYNOTE-028, KEYNOTE-158, and CheckMate358 have demonstrated significant clinical benefits of PD-1/PD-L1 inhibitors in cervical cancer (3). Based on this data, in June 2018, the U.S. Food and Drug Administration (FDA) approved pembrolizumab for the treatment of advanced cervical cancer that has progressed after chemotherapy and expresses PD-L1 (4). Furthermore, some small molecule PD-1 and PD-L1 inhibitors have also emerged and demonstrated certain effects in preclinical experiments (5). These drugs, generated by blocking the immune inhibitory checkpoint through PD1 and PD-L1, have produced unprecedented and durable responses in various cancer types. However, one of the major challenges in ICB therapy is overcoming intrinsic and acquired resistance mechanisms, for which various attempts, such as combination therapy with multiple drugs, have been made. Among them, degradation therapy may be one of the better approaches. However, it is still challenging to develop targeted immune checkpoint degraders using the existing small molecule trimeric PROTAC (Proteolysis-targeting chimera) degrader technology. Mengyuan Dai et al. have targeted PD-1 and PD-L1 using peptides, but the instability of linear peptides brings uncertainty to their future clinical translation (6). On the other hand, a modified stapled peptide PROTAC, with a simple alpha-helical structure, has shown stable and efficient degradation effects. In comparison with the PD-L1 inhibitor BMS8, it demonstrates a more potent inhibition of PD-L1 (Shi et al.). This makes immune checkpoint degraders (ICD) one of the potential new types of tumor immunotherapy in the future.

## Author contributions

RT: Writing – review & editing, Writing – original draft. ML: Writing – review & editing, Investigation. YZ: Writing – review & editing, Conceptualization, Resources. RL: Writing – review & editing, Writing – original draft.
